# Minority K65R Variants and Early Failure of Antiretroviral Therapy in HIV-1–infected Eritrean Immigrant

**DOI:** 10.3201/eid1710.110592

**Published:** 2011-10

**Authors:** Vineeta Bansal, Karin J. Metzner, Barbara Niederöst, Christine Leemann, Jürg Böni, Huldrych F. Günthard, Jan S. Fehr

**Affiliations:** University Hospital Zurich, Zurich, Switzerland (V. Bansal, K.J. Metzner, B. Niederöst, C, Leeman, H.F. Günthard, J.S. Fehr);; University of Zurich, Zurich (J. Böni)

**Keywords:** retroviruses, HIV, antiretroviral therapy, ART, drug-resistant HIV-1 minority variants, K65R, HIV-1 subtype C, resource-limited setting, early virological failure, letter

**To the Editor:** Genotypic drug resistance testing before initiation of first-line antiretroviral therapy (ART) is recommended to detect drug-resistant viruses and to avoid treatment failure caused by preexisting drug-resistant viruses (*1*). However, standard resistance testing cannot detect drug-resistant HIV-1 minority variants unless they represent 20%–25% of the population (*2*). Approximately 15% of those who underwent seroconversion in the acute phase in industrialized settings harbor drug-resistant HIV-1 minority variants, while standard resistance testing did not detect drug-resistant viruses in those patients (*3*). We report the case of a treatment-naive HIV-1–infected patient with early treatment failure because of preexisting minority K65R-harboring HIV-1 variants.

A 32-year-old immigrant to Switzerland from Eritrea with a recently diagnosed HIV-1 subtype C infection was seen at University Hospital, Zurich. On the basis of the low CD4+ T-cell count of 69 cells/µL (15%) and high HIV-1 viral load of 980,000 copies/mL plasma, we started directly observed ART with tenofovir and emtricitabine plus nevirapine. Genotypic resistance testing showed no evidence of resistance. Within the first 4 weeks of ART, the viral load decreased to 540; however, 4 weeks later it increased to 15,000, and then 12 days later to 71,000 HIV-1 RNA copies/mL (Figure). Resistance testing at this time revealed the reverse transcriptase (RT) mutations K65R, K103N, and M184V, which confer resistance to all prescribed drugs. ART was changed to lamivudine/zidovudine, darunavir/ritonavir, and etravirine, and subsequently viremia decreased and remained undetectable.

**Figure:**
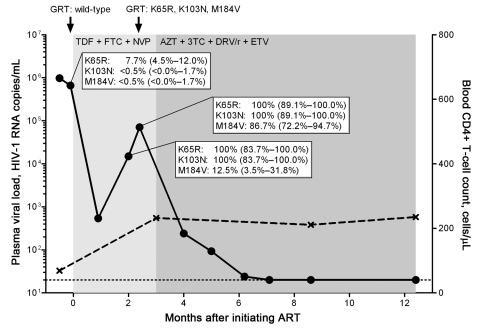
Kinetics of viremia, CD4+ T-cell count, and drug resistance mutations in a treatment-naive person from Eritrea, infected with HIV-1 subtype C, who was experiencing early antiretroviral therapy (ART) failure. Viral load (circles) was measured by using the Cobas AmpliPrep TaqMan HIV-1 test version 2.0 (Roche Diagnostics, Rotkreuz, Switzerland) with a detection limit of 20 HIV-1 RNA copies/mL plasma (dotted line). CD4+ T-cell count is depicted in crosses. Genotypic resistance testing (GRT) based on population sequencing was performed at time points indicated by black arrows. Duration of different ART regimens is shown in shades of gray. Part of the HIV-1 reverse transcriptase (codons 52–218) gene was cloned and sequenced at time points before ART and during virologic failure: n = 222 clones at –2 days before ART, n = 24 clones, and n = 38 clones during virologic failure, respectively. The dynamics of the selection of the K65R, K103N, and M184V mutations are depicted by percentages and 95% confidence intervals. TDF, tenofovir; FTC, emtricitabine; NVP, nevirapine; AZT, zidovudine; 3TC, lamivudine; DRV/r, darunavir in combination with ritonavir; ETV, etravirine.

We hypothesized that preexisting drug-resistant HIV-1 minority variants might have caused this early treatment failure. Thus, we performed clonal analysis of the RT gene before and during ART. At baseline, 17/222 clones (7.7%) carried the K65R mutation, synonymous to ≈51,000 HIV-1 RNA copies. Later, the K65R mutation was comprised in all clones. Further preexisting drug-resistance mutations have been detected in single, K65 wild-type, separate clones: K70R, V106A, and V108I. However, neither the K103N nor the M184V mutation was detected in any of those 222 clones, but both mutations were rapidly selected during early treatment failure.

Thus, the presence of the K65R mutation in a substantial fraction of the virus population and the rapid acquisition of the K103N and M184V mutations led eventually to early treatment failure. We cannot formally rule out that K103N mutants were present before ART at a very low frequency <0.5% and that K65R/K103N double mutants were potentially selected. The M184V mutation was acquired later because only 12.5% of K65R/K103N viruses carried the M184V mutation at the first time point during treatment failure.

Because of the considerable absolute number of less replication-competent K65R-harboring viruses in the patient, we assume that this variant has been transmitted. The additional presence of isolated, nonnucleoside RT inhibitor (NNRTI)/nucleoside RT inhibitor (NRTI) resistance mutations is another indication for the transmission of drug-resistant viruses, although they and the K65R mutation were not found in the same viral genomes. Presumably, the index patient was treated with nevirapine, stavudine, and lamivudine, the commonly prescribed first-line ART in resource-limited settings (*4*).

The prevalence of K65R-harboring drug-resistant HIV-1 minority variants is not negligible in treatment-naive patients. We have shown that 2.7% of HIV-1–infected patients, mainly those infected with HIV-1 subtype B, harbor the K65R mutation as a minority variant (*5*), which is comparable with the prevalence of minority K65R-harboring variants (4%) in patients from South Africa who are infected with HIV-1 subtype C (*6*). A meta-analysis showed that limited or unavailable HIV-1 RNA monitoring in combination with ART regimens with a low genetic barrier to resistance in resource-limited settings is associated with high rates of NNRTI/NRTI resistance in patients for whom ART fails (*7*). Although ritonavir-boosted protease inhibitors containing ART regimens have a higher genetic barrier to resistance and would be preferable in such settings, they are generally not part of first-line therapy in resource-limited settings. Thus, proper monitoring and drug-resistance testing would be desirable when using NNRTI-based ART regimens. Moreover, it could be beneficial to apply sensitive assays for the detection of drug-resistant HIV-1 minority variants in clinical practice. However, clinical cut-off levels for those minority variants need to be defined.

Immigration from resource-limited settings is increasing, and data suggest that minority variants harboring the K65R mutation are quite prevalent in those infected with HIV-1 subtype C, who are treatment naive (*6*). The case described here demonstrates that these drug-resistant HIV-1 minority variants can quickly accumulate further drug-resistance mutations and lead to early treatment failure, especially in the context of an ART regimen with low genetic barriers to resistance. This case was the first among immigrants treated in our center, but, given the potential for considerable transmission rates of resistance in countries that lack virologic monitoring, this mutation could become a larger problem.
